# The impact of EpCAM expression on response to chemotherapy and clinical outcomes in patients with epithelial ovarian cancer

**DOI:** 10.18632/oncotarget.17871

**Published:** 2017-05-15

**Authors:** Shingo Tayama, Takeshi Motohara, Dashdemberel Narantuya, Chenyan Li, Koichi Fujimoto, Isao Sakaguchi, Hironori Tashiro, Hideyuki Saya, Osamu Nagano, Hidetaka Katabuchi

**Affiliations:** ^1^ Department of Obstetrics and Gynecology, Faculty of Life Sciences, Kumamoto University, Chuo-Ku, Kumamoto 860-8556, Japan; ^2^ Department of Maternal-Newborn Nursing, Kumamoto University, Chuo-Ku, Kumamoto 860-0976, Japan; ^3^ Division of Gene Regulation, Institute for Advanced Medical Research, School of Medicine, Keio University, Shinjuku-Ku, Tokyo 160-8582, Japan

**Keywords:** ovarian cancer, cancer stem cell, EpCAM, chemoresistance, prognosis

## Abstract

Epithelial ovarian cancer is a highly lethal malignancy; moreover, overcoming chemoresistance is the major challenging in treating ovarian cancer patients. The cancer stem cell (CSC) hypothesis considers CSCs to be the main culprits in driving tumor initiation, metastasis, and resistance to conventional therapy. Although growing evidence suggest that CSCs are responsible for chemoresistance, the contribution of CSC marker EpCAM to resistance to chemotherapy remains unresolved.

Here we have demonstrated that ovarian cancers containing high levels of EpCAM have a significantly much lower probability of achieving overall responsive rates after first-line chemotherapy. In addition, multivariate analysis revealed that EpCAM expression is an independent risk factor for chemoresistance, indicating that EpCAM expression is a predictive biomarker of chemotherapeutic response. Consistent with these clinical observations, *in vitro* assays, we found that the subpopulation of EpCAM-positive ovarian cancer cells shows a significantly higher viability compared with EpCAM-negative cells in response to cisplatin treatment by preventing chemotherapy-induced apoptosis, which is regulated by EpCAM-Bcl-2 axis. Furthermore, in an *in vivo* mouse model, platinum agents preferentially eliminated EpCAM-negative cells in comparison with EpCAM-positive cells, suggesting that the remaining subpopulation of EpCAM-positive cells contributes to tumor recurrence after chemotherapy. Finally, we also found that an increased expression of EpCAM is associated with poor prognosis in ovarian cancer patients.

Our findings highlight the clinical significance of EpCAM in the resistance to chemotherapy and provide a rationale for EpCAM-targeted therapy to improve chemoresistance. Targeting EpCAM should be a promising approach to effectively extirpate the CSCs as the putative root of ovarian cancer.

## INTRODUCTION

Epithelial ovarian cancer is the leading cause of death from gynecological malignancies [1]. Because the majority of ovarian cancer patients are diagnosed at an advanced stage [2], the clinical outcomes for ovarian malignancies are poor even after treatment with extirpative surgery and intensive chemotherapy [3]. Even though the ovarian cancer may respond to first-line platinum-based chemotherapy, most tumors undergo relapse that is involved in chemoresistant residual cancer cells [1, 4]. Thus, an understanding of the molecular events underlying the resistance to chemotherapy has the potential to have a significant impact on the outcomes of ovarian cancer patients [5].

Growing evidence indicates that human cancers comprise hierarchies of cells sustained by cancer stem cells (CSCs) [6, 7]. CSCs possess the ability to self-renew and to undergo multilineage differentiation and are inherently responsible for tumor metastasis and resistance to chemotherapy [8–11]. Although ovarian CSCs have not been completely characterized, this small population of cancer cells is believed to play a key role in chemoresistance and relapse of this fatal disease [12]. Hence, elucidating the molecular mechanisms that control chemoresistance in relation to the biology of ovarian CSCs may provide potential molecular targets for the treatment of ovarian cancer.

Epithelial cell adhesion molecule (EpCAM) [13, 14], initially discovered as a predominant antigen in human colon cancer [15], is a type I transmembrane glycoprotein that is expressed on subsets of normal epithelia [14] and numerous stem cells [16] and is also overexpressed in a heterogeneous manner in some epithelial cancers [17], including ovarian cancer [18]. Even though the detailed function of EpCAM is still largely unknown, recent evidence suggests that the role of EpCAM is not limited to cell adhesion, but it is correlated with cell proliferation, differentiation, and cellular signaling [13, 15, 19]. Furthermore, over the past decade, EpCAM has been rediscovered as a CSC marker in colon [20], breast [21], hepatocellular [22], and pancreatic cancers [23], which makes it a potential molecular target for novel cancer therapy. We previously identified a subpopulation of EpCAM-positive cancer cells as candidates for CSCs in established mouse ovarian tumors generated by transduction of defined genetic alterations. In a limiting dilution assay, EpCAM-positive cancer cells isolated from hierarchically organized ovarian tumors showed highly tumorigenic properties in comparison with EpCAM-negative cells. Furthermore, we also found that EpCAM-positive cells possess the ability to give rise to less tumorigenic EpCAM-negative cells [24]. In addition, although it is reported that EpCAM is correlated with chemoresistance in several types of epithelial cancer [7, 25, 26], the association between EpCAM and chemoresistance in respect to the biology of ovarian CSCs has remained obscure. Taken together, these findings led us to hypothesize that EpCAM-positive ovarian cancer cells might play a key role in tumor resistance to chemotherapy as one of the most significant features of CSCs.

The present study was designed to elucidate the role of EpCAM in tumor resistance to chemotherapy and the potential relevance of EpCAM expression to the clinical outcomes of patients with ovarian cancer.

## RESULTS

### Correlation between EpCAM expression pattern and clinicopathological characteristics in patients with ovarian cancer

**Figure 1:**
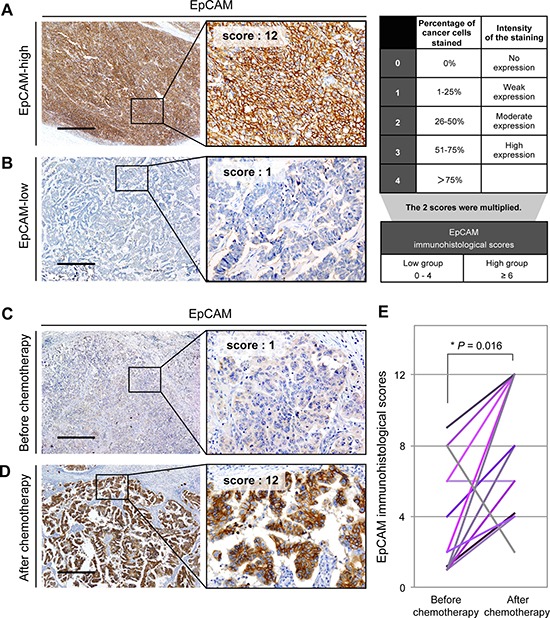
EpCAM expression is increased in ovarian cancer tissues obtained after platinum-based chemotherapy (**A**) A representative immunohistochemical staining pattern for EpCAM in the EpCAM-high group. EpCAM-high group was defined as a total score ≥ 6 (Scale bar: 500 μm). (**B**) A representative immunohistochemical staining pattern for EpCAM in the EpCAM-low group. EpCAM-low group was defined as a total score 0 to 4 (Scale bar: 500 μm). (**C**) Immunohistochemical analysis with anti-EpCAM antibody of ovarian cancer tissues from patients treated without preoperative chemotherapy (Scale bar, 500 μm). (**D**) Immunohistochemical analysis with anti-EpCAM antibody of ovarian cancer tissues from patients treated with adjuvant chemotherapy (Scale bar, 500 μm). (**E**) Statistical analysis of the immunohistochemical scores of EpCAM in 13 paired samples. The scores of EpCAM expression are significantly higher in ovarian cancer tissues from patients treated with chemotherapy than in those from matched patients treated without chemotherapy (Wilcoxon signed-rank test, *P = 0.016).

The clinical significance of EpCAM was evaluated by immunohistochemical analysis using primary ovarian cancer tissues from 168 patients. Based on the scoring of immunohistochemical staining, 97 (57.7%) cases belonged in the EpCAM-high group (Figure 1A), and 71 (42.3%) cases to the EpCAM-low group (Figure 1B). The relationship between EpCAM expression and the clinicopathological features of the 168 patients is shown in Table 1. The median age at diagnosis for patients in the EpCAM-high group was 55.0 years (range, 27 to 87 years) and was similar to in the EpCAM-low group (median, 51 years; range, 22 to 79 years) (*P* = 0.331). Clinicopathological characteristics, such as histological type, FIGO stage, tumor marker CA125, and tumor size did not significantly differ between the EpCAM-high and -low groups. Systematic chemotherapy was given to 145 patients (86.3%) as clinically indicated in accordance with standard practices, and almost all patients received first-line platinum-based chemotherapy. There were no significant differences in the number of cycles of chemotherapy between EpCAM-high and -low groups (*P* = 0.398) (Table 1).

**Table 1:** Association between EpCAM expression pattern and clinicopathological features in patients with ovarian cancer

Patient characteristic	Total (*n* = 168)	EpCAM		*P* value
		high (*n* = 97)	low (*n* = 71)	
Median age (range)	55.0 (22–87)	55.0 (27–87)	51.0 (22–79)	0.331
Menopause status				
pre	57 (33.9%)	29 (29.9%)	28 (39.4%)	0.197
post	111 (66.1%)	68 (70.1%)	43 (60.6%)	
Median BMI (range)	22.2 (15.3–32.7)	22.4 (15.9–32.4)	21.9 (15.3–32.7)	0.385
Histological type				
serous	75 (44.6%)	43 (44.3%)	32 (45.1%)	0.111
clear cell	23 (13.7%)	9 (9.3%)	14 (19.7%)	
endometrioid	28 (16.7%)	17 (17.5%)	11 (15.5%)	
mucinous	20 (11.9%)	16 (16.5%)	4 (5.6%)	
other	22 (13.1%)	12 (12.4%)	10 (14.1%)	
FIGO stage				
I	63 (37.5%)	32 (33.0%)	31 (43.7%)	0.446
II	17 (10.1%)	12 (12.4%)	5 (7.0%)	
III	59 (35.1%)	36 (37.1%)	23 (32.4%)	
IV	29 (17.3%)	17 (17.5%)	12 (16.9%)	
CA125				
< 500 U/mls	87 (51.8%)	48 (49.5%)	38 (53.5%)	0.700
≥ 500 U/ml	81 (48.2%)	49 (50.5%)	33 (46.5%)	
Tumor size				
< 10 cm	77 (45.8%)	46 (47.4%)	31 (43.7%)	0.629
≥ 10 cm	91 (54.2%)	51 (52.6%)	40 (56.3%)	
Residual tumor size				
0 mm (complete surgery)	112 (66.7%)	63 (65.0%)	49 (69.0%)	0.127
1–10 mm (optimal surgery)	17 (10.1%)	7 (7.2%)	10 (14.1%)	
> 10 mm (suboptimal surgery)	39 (23.2%)	27 (27.8%)	12 (16.9%)	
First-line chemotherapy				
Platinum based chemotherapy	144 (85.7%)	83 (85.6%)	61 (85.9%)	0.688
Other regimen	1 (0.6%)	1 (1.0%)	0 (0.0%)	
No adjuvant therapy	23 (13.7%)	13 (13.4%)	10 (14.1%)	
No. of cycles of chemotherapy				
< 2	137 (81.5%)	77 (79.4%)	60 (84.5%)	0.398
≥ 3	31 (18.5%)	20 (20.6%)	11 (15.5%)	

### Increased expression of EpCAM in ovarian cancer tissues obtained after platinum-based chemotherapy

To explore the clinical relevance of EpCAM in ovarian cancer patients, we compared the EpCAM expression among the 13 primary samples of ovarian cancer treated without preoperative chemotherapy to that of the samples taken from the same patients underwent secondary debulking surgery after adjuvant platinum-based chemotherapy. Representative immunohistochemical staining patterns for EpCAM of both before and after chemotherapy are shown in Figure 1C, 1D. Immunohistochemical analysis revealed that the staining intensity of EpCAM, and the relative area occupied by EpCAM-positive cancer cells were significantly higher in tumors of patients who received platinum-based chemotherapy than in those of matched patients who did not (*P* = 0.016; Figure 1E). These findings suggested that EpCAM-positive cancer cells are clinically involved in resistance to platinum-based chemotherapy.

### EpCAM expression as an independent risk factor for resistance to chemotherapy in patients with ovarian cancer

To further investigate whether a causal relationship exists between EpCAM expression and chemotherapeutic response, we analyzed 52 patients with ovarian cancer who had undergone platinum-based chemotherapy, except for cases achieving complete surgery, defined as no visible residual tumors. The correlation between EpCAM expression and response to first-line chemotherapy is shown in Table 2. Complete response (CR) was achieved in 7 cases (21.9%) in the EpCAM-high group and in 6 cases (30.0%) in the EpCAM-low group. Partial response (PR) was observed in 13 cases in the EpCAM-high group (40.6%) and 12 cases (60.0%) in the EpCAM-low group. There were significant differences in response to chemotherapy between EpCAM-high and -low groups (*P* = 0.007). Notably, EpCAM-high group had significantly lower overall response rates (ORR: CR and PR) after first-line treatment when compared with the EpCAM-low group (62.5% vs. 90.0%, *P* = 0.030) (Table 2).

**Table 2:** Response to chemotherapy in patients with ovarian cancer

Response	Total (*n* = 52)	EpCAM		*P* value
		high (*n* = 32)	low (*n* = 20)	
Complete response (CR)	13	7 (21.9%)	6 (30.0%)	0.007
Partial response (PR)	25	13 (40.6%)	12 (60.0%)	
Stable disease (SD)	2	0 (0.0%)	2 (10.0%)	
Progressive disease (PD)	12	12 (37.5%)	0 (0.0%)	
Overall response rate (ORR: CR+PR)	38	20 (62.5%)	18 (90.0%)	0.030

To evaluate the factors that influenced chemotherapeutic response of ovarian cancer, univariate and multivariate analysis of various clinicopathological factors in relation to ORR was performed (Table 3). As a result, EpCAM expression was identified as a significant predictor of the chemoresistance in ovarian cancer patients according to the univariate logistic regression analysis (OR, 5.40; 95% confidence interval [CI], 1.06–27.47; *P* = 0.042) and the multivariate Cox proportional hazards model (OR, 11.12; 95% CI, 1.66–74.41; *P* = 0.013). These data indicate that immunohistochemical expression of EpCAM is an independent risk factor for tumor resistance to chemotherapy in patients with ovarian cancer.

**Table 3:** Odds ratios (ORs) using univariate and multivariate logistic regression analysis for overall response rate (ORR)

Variable		No. at risk	event	Univariate analysis			Multivariate analysis		
				OR	95% CI	*P* value	OR	95% CI	*P* value
Age	< 50	14	3	Reference					
	≥ 50	38	11	1.49	0.35–6.41	0.589			
CA125	< 500 U/ml	12	4	Reference					
	≥ 500 U/ml	40	10	0.67	0.17–2.70	0.570			
Histological type	serous	36	7	Reference					
	non serous	16	7	3.22	0.89–11.67	0.075			
FIGO stage	III	33	5	Reference			Reference		
	IV	19	9	5.04	1.36–18.68	0.016	9.68	1.97–47.65	0.005
No. of cycles of chemotherapy	< 2	33	8	Reference					
	≥ 3	19	6	1.44	0.41–5.05	0.567			
Tumor size	< 10 cm	34	8	Reference					
	≥ 10 cm	18	6	1.63	0.46–5.73	0.450			
Surgical debulking status	Optimal surgery	16	3	Reference					
	Suboptimal surgery	36	11	1.91	0.45–8.06	0.380			
EpCAM expression	Low	20	2	Reference			Reference		
	High	32	12	5.40	1.06–27.47	0.042	11.12	1.66–74.41	0.013

### Tumor resistance to platinum chemotherapeutic agents in a subpopulation of EpCAM-positive ovarian cancer cells *in vitro*

**Figure 2:**
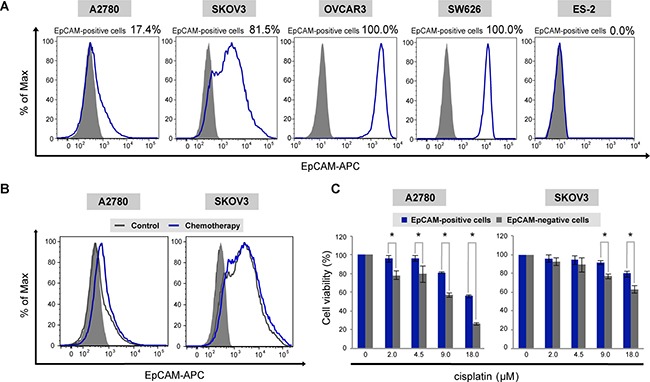
The subpopulation of EpCAM-positive ovarian cancer cells is associated with chemoresistance to platinum chemotherapeutic agents *in vitro* (**A**) Flow cytometric analysis of ovarian cancer cell lines with APC-conjugated antibody to EpCAM. (**B**) Flow cytometric analysis of EpCAM expression in A2780 and SKOV3 cells. Treatment with cisplatin resulted in enhanced expression of EpCAM in residual cancer cells as compared with untreated cells. (**C**) Chemosensitivity assay in FACS-sorted EpCAM-positive and EpCAM-negative cancer cells. Cells were subjected to MTS assay to assess the viability in the presence of cisplatin. Sorted EpCAM-positive cancer cells showed significantly higher viability compared with sorted EpCAM-negative cancer cells (*P < 0.01).

Given that EpCAM was clinically associated with chemoresistance to platinum-based chemotherapy, we next examined the relevance of chemoresistance in a subpopulation of EpCAM-positive cells in *in vitro* assays using ovarian cancer cell lines. To investigate the heterogeneity of EpCAM expression in each cell line, we screened the EpCAM expression by flow cytometric analysis. Immunofluorescence labeling of ovarian cancer cells with anti-EpCAM antibody showed various expression patterns of EpCAM (Figure 2A). Among these cell lines, A2780 and SKOV3 cells consist of two subpopulations, EpCAM-positive and -negative cells; therefore, we used these two cell lines with a hierarchically organized cell population for further studies.

To investigate whether the subpopulation of EpCAM-positive cells is involved in resistance to platinum chemotherapeutic agents, ovarian cancer cells were exposed to cisplatin *in vitro*. Flow cytometric analysis revealed that treatment with cisplatin resulted in enhanced expression of EpCAM in residual cells as compared to untreated cells (Figure 2B). In addition, FACS-sorted EpCAM-positive cells showed significantly higher viability compared with sorted EpCAM-negative cells in the presence of platinum agents in MTS assay (Figure 2C), suggesting that the subpopulation of EpCAM-positive cells is involved in tumor resistance to the platinum chemotherapeutic agents.

### Chemoresistance of EpCAM-positive ovarian cancer cells in an *in vivo* ovarian cancer mouse model

**Figure 3:**
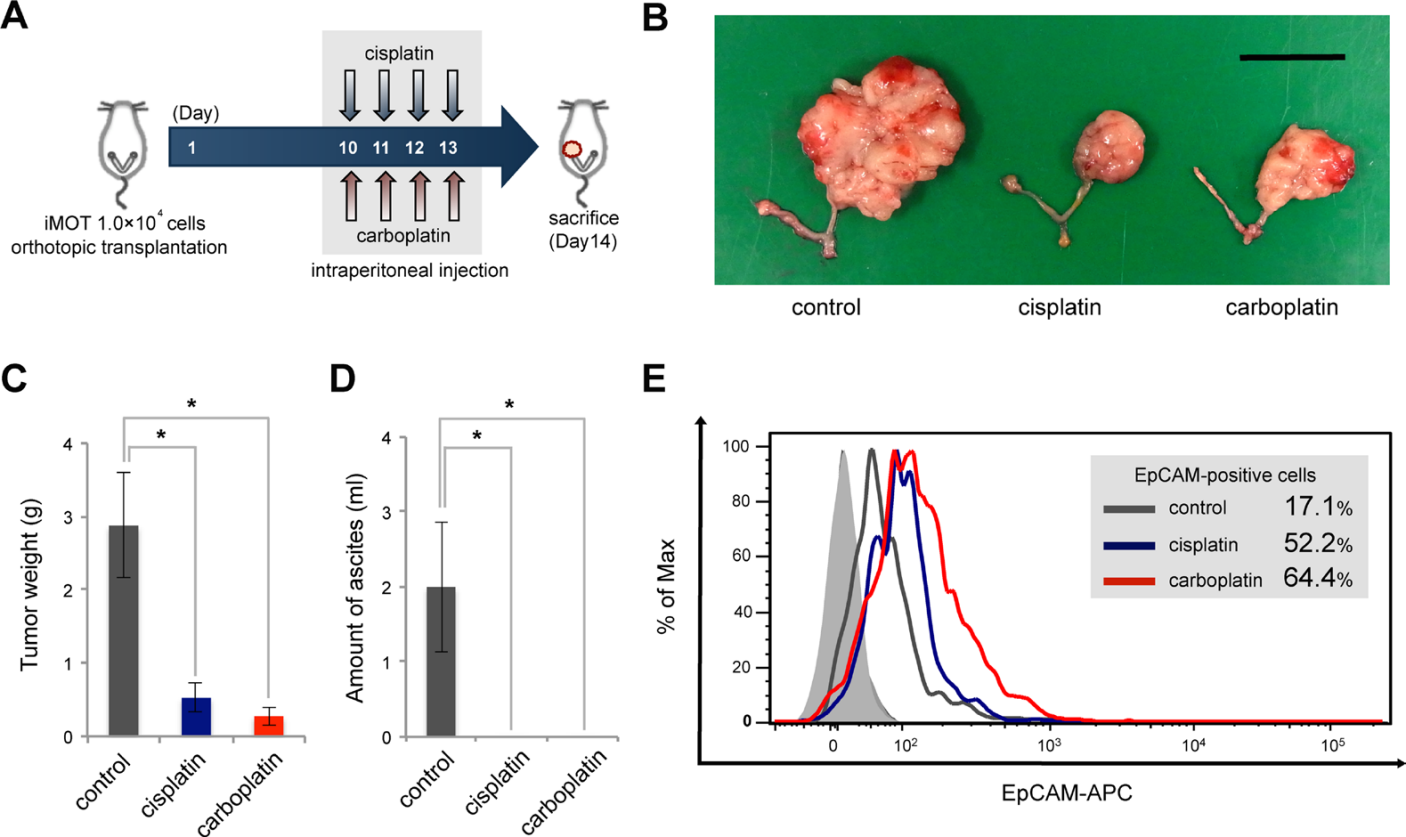
EpCAM-positive ovarian cancer cells are correlated with tumor resistance to chemotherapy in an *in vivo* mouse model (**A**) Schema of platinum chemotherapeutic treatments in an orthotopic ovarian tumor mouse model. iMOT cells (1.0 × 104 cells) were transplanted into the left ovarian bursa of 7-week-old female C57BL/6 mice. Ovarian tumor-bearing mice received intraperitoneal injections of cisplatin, carboplatin, or PBS on days 10, 11, 12, 13 after orthotopic transplantation, respectively. (**B**) Macroscopic appearance of mouse ovarian tumors treated with or without platinum agents at 14 days after orthotopic cell transplantation (Scale bar, 2 cm). (**C**) Tumor weight determined at 14 days after cell injection in each group. Quantitative data are presented as mean ± SD for five mice (*P < 0.01). (**D**) Ascitic volume evaluated at 14 days after cell transplantation in each group. Quantitative data are presented as mean ± SD for five mice (*P < 0.01). (**E**) Flow cytometric analysis of EpCAM expression in mouse ovarian tumors treated with or without platinum agents. Platinum agents induced substantial enrichment of the EpCAM-positive cells in mouse ovarian tumors.

We previously established induced mouse ovarian tumor-initiating (iMOT) cells by siRNA-mediated transient knockdown of p53 followed by retroviral transduction of c-Myc and K-Ras oncogenes[24]. To evaluate the *in vivo* relevance of resistance to platinum chemotherapy in EpCAM-positive cancer cells, iMOT cells were orthotopically transplanted into immunocompetent recipient mice, and recipient mice bearing ovarian tumors received intraperitoneal cisplatin or carboplatin treatment (Figure 3A). Treatment with either drug caused significant tumor shrinkage in comparison with the control group (Figure 3B–3D); however, substantial enrichment of the EpCAM-positive cells was observed (Figure 3E). These findings indicated that platinum-based chemotherapy preferentially eliminates EpCAM-negative cancer cells, and the remaining subpopulation of EpCAM-positive cancer cells associated with chemoresistance might be responsible for tumor recurrence after such treatment.

### Resistance to chemotherapy-induced apoptosis in a subpopulation of EpCAM-positive ovarian cancer cells

**Figure 4:**
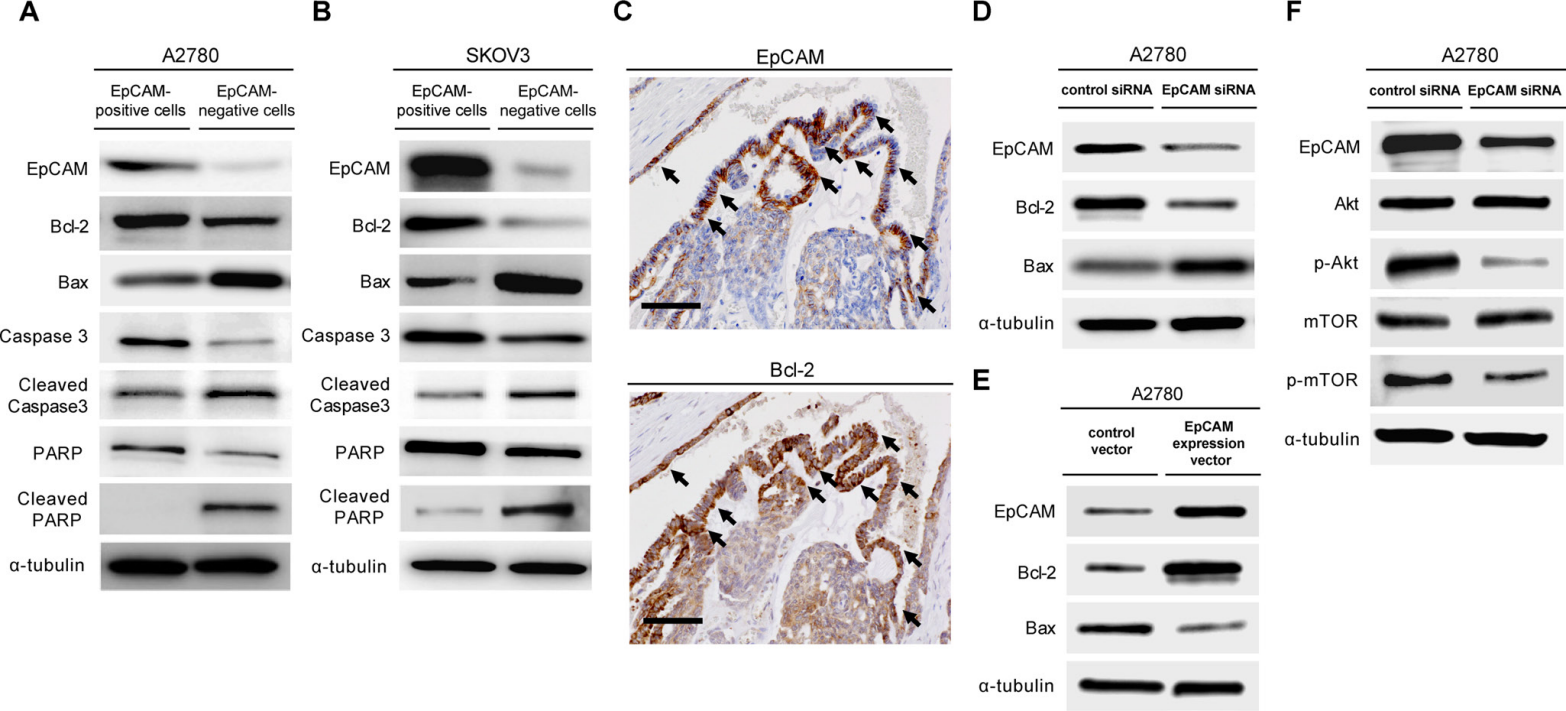
The subpopulation of EpCAM-positive ovarian cancer cells prevents platinum anticancer drug-induced apoptosis To compare the anti-apoptotic ability of EpCAM-positive and EpCAM-negative cancer cells, we sorted EpCAM-positive and EpCAM-negative cells from the A2780 ovarian cancer cell line (**A**) and SKOV3 cell line (**B**). FACS-sorted EpCAM-positive and -negative cells were treated with18 μM cisplatin for 24 h, and the expression of apoptosis-associated proteins, including bcl-2, bax, caspase-3, and poly (ADP-ribose) polymerase (PARP), were examined by western blot analysis. (**C**) Representative immunohistochemical EpCAM and Bcl-2 staining in serial sections of ovarian cancer specimens. Bcl-2 was mainly detected EpCAM-positive cancer cells (arrows) (Scale bar: 100 μm). (**D**) Immunoblot analysis of EpCAM, Bcl-2, and Bax in A2780 cells transfected EpCAM or control siRNAs. (**E**) Immunoblot analysis of EpCAM, Bcl-2, and Bax in A2780 cells transduced overexpressing EpCAM (pCMV6-EpCAM expression vector) and their respective controls (pCMV6 empty vector). (**F**) A2780 cells transfected with EpCAM or control siRNAs were subjected to immunoblot analysis with indicated antibodies.

To further analyze the molecular mechanisms underlying chemoresistance in a subpopulation of EpCAM-positive cancer cells, we investigated the effect of EpCAM expression on platinum anticancer drug-induced apoptosis. FACS-sorted EpCAM-positive and -negative cells were exposed to cisplatin *in vitro*, and the expression of apoptosis-related proteins were examined by Western blot analysis. As a result, the expression of anti-apoptotic Bcl-2 in EpCAM-positive cells was higher than that in EpCAM-negative cells, whereas the expression of pro-apoptotic bax in EpCAM-positive cells was lower than that in EpCAM-negative cells. In addition, activation of caspase-3 and PARP fragmentation, which are markers indicative of apoptosis, in EpCAM-positive cells were lower than that in EpCAM negative cells (Figure 4A, 4B). To further examine whether EpCAM expression correlates with Bcl-2 expression in ovarian cancer tissues, serial sections of ovarian cancer specimens were stained with EpCAM and Bcl-2 antibodies. Immunohistochemical analysis revealed that Bcl-2 expression is detected mainly in EpCAM-positive cancer cells, confirming their association with Bcl-2 expression in ovarian cancer (Figure 4C). Taken together, these results suggested that a subpopulation of EpCAM-positive cells is correlated with resistance to chemotherapy-induced apoptosis, which is regulated by EpCAM-Bcl-2 axis.

Next, the effect of siRNA-mediated EpCAM knockdown on the expression of apoptosis-related proteins was assessed. Western blot analysis revealed that siRNA-mediated EpCAM knockdown affects apoptosis in A2780 cells by downregulating Bcl-2 expression and upregulating Bax expression (Figure 4D). Furthermore, successful transfection of EpCAM overexpression in A2780 cells led to upregulation of Bcl-2 expression and concomitant downregulation of Bax expression (Figure 4E).

Cell apoptosis is controlled by a complex network of proliferation and survival genes. To date, several studies have linked the PI3K/Akt/mTOR signaling pathway to resistance to chemotherapy [27]. To investigate whether EpCAM expression correlates with PI3K/Akt/mTOR signaling pathway, we assessed Akt, p-Akt, mTOR, and p-mTOR exressions in A2780 cells after siRNA-mediated EpCAM knockdown. Our results show that p-Akt and p-mTOR expressions are downregulated in A2780 cells transfected with a siRNA specific for EpCAM (Figure 4F), suggesting that EpCAM expression is closely associated with PI3K/Akt/mTOR signaling pathway and leads to resistance to chemotherapy.

### Prognostic impact of EpCAM expression in patients with ovarian cancer

**Figure 5:**
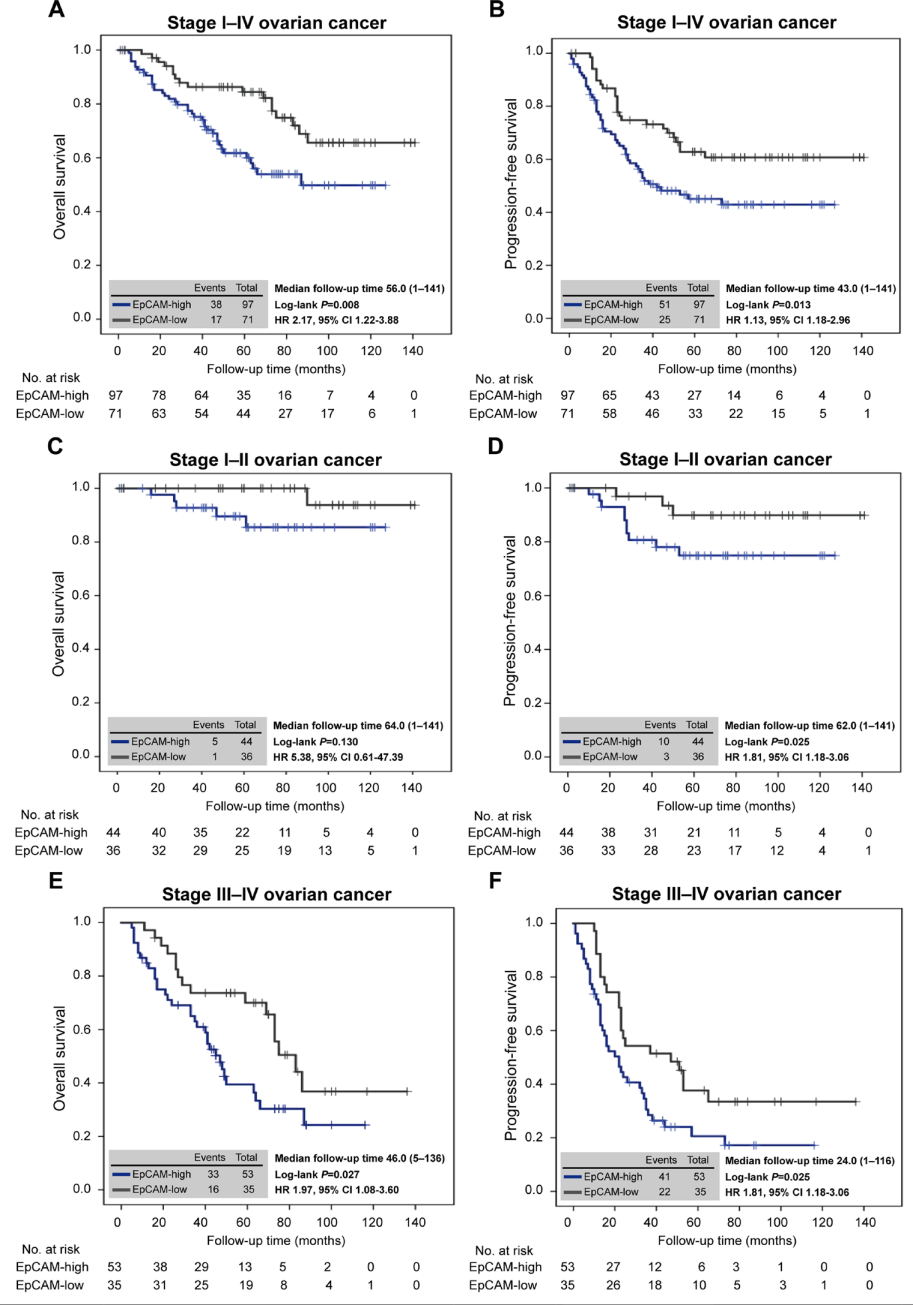
EpCAM expression predicts ovarian cancer survival (**A**) Kaplan–Meier analysis of overall survival in patients with stage I–IV ovarian cancer according to the expression of EpCAM. There were significant differences in overall survival between the EpCAM-high and -low groups (HR, 2.17; 95% CI, 1.22–3.88; *P* = 0.008). (**B**) Kaplan–Meier analysis of progression-free survival in patients with stage I–IV ovarian cancer according to the expression of EpCAM. Progression-free survival was significantly different between the EpCAM-high and -low groups (HR, 1.13; 95% CI, 1.18–2.96; *P* = 0.013). (**C**) Kaplan–Meier analysis of overall survival in patients with stage I–II ovarian cancer according to the expression of EpCAM. There were no significant differences in overall survival between the EpCAM-high and -low groups (HR, 5.38; 95% CI, 0.61–47.39; *P* = 0.130). (**D**) Kaplan–Meier analysis of progression-free survival in patients with stage I–II ovarian cancer according to the expression of EpCAM. Progression-free survival was significantly different between the EpCAM-high and -low groups (HR, 1.81; 95% CI, 1.18–3.06; *P* = 0.025). (**E**) Kaplan–Meier analysis of overall survival in patients with stage III–IV ovarian cancer according to the expression of EpCAM. Overall survival was significantly different between the EpCAM-high and EpCAM-low groups (HR, 1.97; 95% CI, 1.08–3.60; *P* = 0.027). (**F**) Kaplan–Meier analysis of progression-free survival in patients with stage III–IV ovarian cancer according to the expression of EpCAM. There were significant differences in progression-free survival between the EpCAM-high and -low groups (HR, 1.81; 95% CI, 1.18–3.06; *P* = 0.025).

Even though previous studies have focused on the potential association of EpCAM with ovarian cancer survival, there is no unified view on this issue[18, 28, 29]. In order to address these unresolved questions, we evaluated the correlation between EpCAM expression and overall survival and progression-free survival in patients with stage I–IV ovarian cancer. Kaplan–Meier analysis demonstrated that the 5-year overall survival rates were 61.7% (95% CI, 51.2–72.3) in the EpCAM-high group and 84.4% (95% CI, 75.5–93.3) in the EpCAM-low group. There were significant differences in overall survival between the EpCAM-high and -low groups for patients with stage I–IV ovarian cancer (Figure 5A). In addition, significant differences were observed in progression-free survival between the EpCAM-high and -low groups for patients with stage I–IV ovarian cancer (Figure 5B).

Moreover, to evaluate the further association with treatment of chemotherapy, patients with ovarian cancer were divided into two subgroups, stage I–II and stage III–IV. In patients with stage I–II ovarian cancer, there were no significant differences in overall survival between the EpCAM-high and -low groups (Figure 5C), whereas progression-free survival was significantly different between the EpCAM-high and -low groups (Figure 5D). In patients with stage III–IV ovarian cancer, overall survival and progression-free survival was statistically significantly shorter in the EpCAM-high group than in the EpCAM-low group (Figure 5E, 5F). These results suggested that EpCAM-positive cancer cells are responsible for poor prognosis with a high degree of chemoresistance.

## DISCUSSION

One of the greatest impediments in improving the clinical outcomes of ovarian cancer has been a lack of understanding of the molecular mechanisms by which residual cancer cells survive after chemotherapy [1, 4, 30, 31]. Recent evidence suggests that the functional and molecular features of CSCs constitute therapeutic opportunities to improve the chemoresistance during cancer treatment [9, 12, 32]. Therefore, it will be crucial to identify CSC markers that predict responsiveness to chemotherapy, which may conduct the development of therapeutic biomarkers aimed at overcoming chemoresistance [33]. In order to improve the outcomes of ovarian cancer patients, uncovering the molecular mechanisms underlying the resistance to chemotherapy and the role of ovarian CSCs is important for the development of novel therapeutic strategies [34].

EpCAM was initially identified as a tumor-associated antigen in 1979 [15] and has been of particular interest because of its high level of expression in a variety of solid cancers [7, 35]. Most importantly, mounting data over recent years have indicated that EpCAM is a useful marker for the isolation of subsets enriched for CSCs [14, 24, 36]. Although reports of cellular and molecular properties of EpCAM in ovarian cancer are limited, EpCAM-positive ovarian cancer cells seem to possess CSC properties [24, 37] and play an important role in resistance to chemotherapy [25].

In the present study, we demonstrated that increased expression of EpCAM contributes to resistance to platinum-based chemotherapy in ovarian cancer patients. Immunohistochemical analysis showed that EpCAM expression is increased significantly in tumor tissues of patients who received platinum-based chemotherapy in comparison with those of the corresponding tumor tissues before chemotherapy, indicating that EpCAM expression is clinically significant and is associated with residual chemoresistant populations that must be present at the end of primary therapy. Intriguingly, we also showed that, in an *in vivo* mouse model, platinum chemotherapeutic agents preferentially kill EpCAM-negative cancer cells in comparison with EpCAM-positive cancer cells, suggesting that the remaining subpopulation of EpCAM-positive cancer cells is inherently responsible for tumor recurrence after such treatment of chemotherapy.

The principal findings of our study were that ovarian cancers containing high levels of EpCAM have a much lower probability of achieving ORR after first-line platinum-based treatment. In addition, multivariate analysis revealed that EpCAM expression in primary tumors was an independent risk factor for chemoresistance, indicating that EpCAM is an important predictive biomarker of chemotherapeutic response. These findings have significant clinical implication, because examination for EpCAM expression in the primary ovarian cancer may estimate chemoresistance in adjuvant chemotherapy.

Consistent with our clinical observations, in *in vitro* assays, we demonstrated that treatment with platinum chemotherapeutic agents enhances the cell surface expression of EpCAM in ovarian cancer cells, and the subpopulation of EpCAM-positive cells showed significantly higher viability compared with EpCAM-negative cells in response to cisplatin treatment by preventing chemotherapy-induced apoptosis. Notably, this study provided new evidence that siRNA-mediated EpCAM knockdown had an effect on apoptosis by downregulating Bcl-2 expression and upregulating Bax expression in ovarian cancer cells. Furthermore, successful transfection of EpCAM overexpression in cancer cells led to upregulation of Bcl-2 expression and downregulation of concomitant Bax expression. These findings indicate that EpCAM regulates cell apoptosis by modulating the expression of apoptosis-related proteins. More importantly, siRNA-mediated EpCAM knockdown resulted in the downregulation of the PI3K/Akt/mTOR signaling pathway, suggesting that EpCAM plays a crucial role in chemoresistance via activation of the PI3K/Akt/mTOR signaling pathway. Taken together, these results indicate that EpCAM and PI3K/Akt/mTOR targeted therapy might be promising strategies for overcoming chemoresistance in patients with ovarian cancer.

Clinical evidence suggested that increased CSCs in a tumor mass contribute to poor prognosis in several types of cancers, including ovarian, colorectal, breast, prostate, and pancreatic cancers [12, 18, 38, 39]. Although previous studies have focused on the relationship between EpCAM expression and survival in patients with ovarian cancer, some controversial results still exist and a consensus has not been reached until now [18, 28]. In order to resolve these unanswered questions, we used Kaplan–Meier analysis to evaluate the capacity of EpCAM expression to predict clinical outcomes of ovarian cancer patients. We demonstrated that an increased expression of EpCAM in primary tumors was correlated with shortened overall and progression-free survival for patients with stage I–IV ovarian cancer, suggesting that EpCAM-positive cancer cells are responsible for poor prognosis with strong ability of chemoresistance.

Considering the still unfavorable prognosis of ovarian cancer patients, the development of novel therapeutic strategies is a prerequisite to eventually achieve better clinical outcomes [4]. In 2009, the European Medicines Agency approved the use of the trifunctional bispecific antibody catumaxomab, which binds to EpCAM and enhances the immunological response against EpCAM-positive cancer cells [40–43]. Notably, in ovarian cancer, EpCAM-positive cancer cells were highly present in malignant ascites of recurrent ovarian cancer patients [42, 44], and the high prevalence of EpCAM-positive cancer cells qualifies this antigen as a prospective target for catumaxomab therapy. Given that EpCAM-positive CSCs are responsible for tumor resistance to chemotherapy, targeting EpCAM might be a promising approach to effectively eradicate ovarian CSCs as the putative root of ovarian cancer [45, 46].

In conclusion, our present study represents the initial report showing EpCAM expression contributes to tumor resistance to chemotherapy in patients with ovarian cancer. These findings provide a rationale for EpCAM-targeted therapy to improve chemoresistance in ovarian cancer cells and sensitize them to currently available treatment. EpCAM may represent not only an important predictor of chemoresistance but also a putative molecular therapeutic target for eradicating ovarian CSCs.

## MATERIALS AND METHODS

### Patients and tissue preparation

We conducted a retrospective cohort study to evaluate the role of EpCAM in resistance to chemotherapy and clinical outcomes in patients with ovarian cancer. We reviewed the medical records and imaging studies of ovarian cancer patients treated from January 2003 to December 2011 at Kumamoto University Hospital. Eligible patients were followed up until December 2014. In the present study, 168 patients who were surgically treated with or without chemotherapy in accordance with standard practices were included. Patients were excluded when they had borderline tumors, non-epithelial tumors, or multiple primary cancers. This study was approved by the institutional review board, and written informed consent was obtained from all patients before treatment.

Ovarian cancer specimens obtained surgically were fixed in 10% buffered formalin, embedded in paraffin wax, and sliced into 4 μm thick sections for histological and subsequent immunohistochemical examinations. Sections were stained with hematoxylin and eosin, and histological diagnosis was performed according to the World Health Organization (WHO) classification of surface epithelial-stromal ovarian tumors [47]. All tumors were staged according to the International Federation of Gynecology and Obstetrics (FIGO) criteria [48].

### Evaluation of immunohistochemical staining

Immunohistochemical analysis was performed as described previously [29, 49]. Briefly, the sections were washed with phosphate-buffered saline (PBS), subjected to antigen retrieval by heating in a microwave in 0.01 M sodium citrate buffer (pH 6.0), and exposed to 3% H_2_O_2_ in methanol before staining with the primary antibody. Immune complexes were detected with use of the avidin–biotin–peroxidase complex (ABC kit, Vector Laboratories, Burlingame, CA, USA) and diaminobenzidine (DAB) substrate (Vector Laboratories), and the sections were counterstained with hematoxylin. EpCAM was detected with the mouse monoclonal EpCAM antibody (B302 [323/a3], Abcam, UK).

The expression level of EpCAM was determined semiquantitatively by reference to previous studies [18, 27, 28]. EpCAM expression scores were evaluated according to the percentage of cancer cells stained (0, 0%; 1, 1%–25%; 2, 26%–50%; 3, 51%–75%; 4, > 75%) and intensity of the staining (0, no staining; 1,weak; 2, moderate; 3, strong), and the 2 scores were then multiplied. As a result, a total score ≥ 6 was defined as “EpCAM-high” group, whereas a total score 0 to 4 was defined as “EpCAM-low” group. Bcl-2 was detected with the rabbit monoclonal Bcl-2 antibody ([E17], ab32124, Abcam, UK). All slides were independently reviewed by three experts who were not informed about the clinical outcomes.

### Cell lines

Human ovarian cancer cell lines, A2780, SKOV3, OVCA3, SW626, and ES-2, were obtained from the American Type Culture Collection (ATCC, Manassas, VA, USA). These cells were maintained in RPMI1640 medium (Wako Pure Chemical Industries. Ltd., Japan) supplemented with 10% fetal bovine serum at 37°C in a 5% CO_2_-containing atmosphere.

### Flow cytometric analysis

Cell sorting and flow cytometric analysis were performed with the use of a fluorescence-activated cell sorter (FACS) (Aria II, BD Biosciences, San Jose, CA, USA). Cells were incubated with allophycocyanin (APC)-conjugated mouse monoclonal antibody EpCAM (HEA-125, Miltenyi Biotec, Bergisch Gladbach, Germany) for 10 min. FACS-sorted EpCAM-positive and -negative cancer cells were used for further analysis.

### Chemosensitivity assay

Cell viability was evaluated with MTS assay according to the manufacturer's protocol (CellTiter 96 Aqueous One Solution Cell Proliferation assay, Promega, Madison, WI, USA). Briefly, cells (3 × 10^3^/100 μL per well) were plated in 96-well flat bottom plates and serum starved overnight. Sorted EpCAM-positive and -negative cancer cells were treated with cisplatin at the indicated concentrations. At 24 h post-drug treatment, 20 μL MTS assay solution was added to each well for 3 h. Absorbance was recorded at 490 nm on an SpectraMax 190 microplate reader (Molecular Devices, Sunnyvale, CA, USA). The percentage of cell survival was defined as the relative absorbance of untreated versus treated cells.

### Immunoblot analysis

Immunoblot analysis was performed as previously described [12]. In brief, equal amounts of FACS-sorted cell lysate protein were subjected to SDS-polyacrylamide gel electrophoresis, transferred to a nitrocellulose membrane, and exposed to anti-EpCAM antibody (B302 (323/A3), Abcam, Cambridge, UK), anti-Bcl-2 (#2872, Cell Signaling Technology, Beverly, MA, USA), anti-Bax (#2772, Cell Signaling Technology, Beverly, MA, USA), anti-PARP (#9542, Cell Signaling Technology, Beverly, MA, USA), anti-Caspase-3 (#9662, Cell Signaling Technology, Beverly, MA, USA), anti-AKT (ab184136; Abcam, Cambridge, UK), anti-phospho-AKT (ab183758; Abcam, Cambridge, UK), anti-mTOR (#2972; Cell Signaling Technology, Beverly, MA, USA), anti-phospho-mTOR (#2971; Cell Signaling Technology, Beverly, MA, USA), and anti-alpha Tubulin (ab4074, Abcam, Cambridge, UK). Immune complexes were visualized by chemiluminescence detection (Pierce Biotechnology, Rockford, IL, USA).

### siRNA transfection

The sequence of EpCAM siRNA duplexes were 5′-UGCUCUGAGCGAGUGAGAATT-3′ and 5′-UUCUCACUCGCUCAGAGCATT-3′. Non-silencing siRNA was used as a negative control. Ovarian cancer cells were transfected with siRNAs for 72 h in the presence of Lipofectamin RNAi MAX reagent (Invitrogen, Tokyo, Japan), according to the manufacturer's protocol.

### Establishement of EpCAM-overexpressing cancer cells

A2780 cells were transfected with a pCMV6 empty vector or pCMV6-EpCAM expression vector (OriGene, Rockville, USA), containing the human EpCAM cDNA, using Lipofectamine 3000 (Invitrogen, Tokyo, Japan) according to the manufacturer's protocol. Transfected cells were selected in a medium with 100 μg/mL ampicillin.

### Animal study

C57BL/6 mice were obtained from CLEA (Tokyo, Japan) and maintained according to institutional guidelines. All animal experiments were performed in accordance with protocols approved by the animal ethics committee of Kumamoto University.

To generate mouse ovarian tumors, iMOT cells [24] (1.0 × 10^4^ cells) were transplanted into the left ovarian bursa of 7-week-old female C57BL/6 mice, and tumor weights and amount of ascites were measured at 14 days after transplantation of iMOT cells. For *in vivo* chemotherapeutic treatments, ovarian tumor-bearing mice received intraperitoneal injections of cisplatin at 5 mg/kg, carboplatin at 10 mg/kg, or PBS on days 10, 11, 12, and 13 after orthotopic injection of iMOT cells, respectively.

### Statistical analysis

Ovarian cancer patients were evaluated for tumor response based upon computed tomography (CT) scan or magnetic resonance imaging (MRI) findings, and disease response was assessed by Response Evaluation Criteria in Solid Tumors (RECIST) criteria [50]. The prognosis of patients was determined according to the cumulative survival rate after treatment. Survival rates were calculated using the Kaplan–Meier method, and differences between curves were assessed with the log-rank test. Correlations between variables were evaluated with the χ^2^ test or Wilcoxon test. Data are presented as mean ± standard deviation (SD) and were analyzed with the Student's *t* test. Univariate and multivariate logistic regression analysis were performed to calculate odds ratios (ORs) using SPSS version 21.0 (IBM Corporation, Armonk, NY, USA). In all analyses, statistical significance was defined as a *P* value of < 0.05.
